# Genome-Wide Identification of Superoxide Dismutase and Expression in Response to Fruit Development and Biological Stress in *Akebia trifoliata*: A Bioinformatics Study

**DOI:** 10.3390/antiox12030726

**Published:** 2023-03-15

**Authors:** Huai Yang, Qiuyi Zhang, Shengfu Zhong, Hao Yang, Tianheng Ren, Chen Chen, Feiquan Tan, Guoxing Cao, Jun Liu, Peigao Luo

**Affiliations:** 1 Key Laboratory of Plant Genetics and Breeding, Sichuan Agricultural University of Sichuan Province, Chengdu 611130, China; 2 College of Forestry, Sichuan Agricultural University, Chengdu 611130, China

**Keywords:** *Akebia trifoliata*, superoxide dismutase, fruit development, biotic stress, expression profile

## Abstract

*Akebia trifoliata* is a newly domesticated perennial fruit tree, and the lack of molecular research on stress resistance seriously affects its genetic improvement and commercial value development. Superoxide dismutase (*SOD*) can effectively eliminate the accumulation of reactive oxygen species (ROS) during the rapid growth of plant organs under biotic and abiotic stresses, maintaining a steady state of physiological metabolism. In this study, 13 *SODs* consisting of two *FeSODs* (*FSDs*), four *MnSODs* (*MSDs*) and seven *Cu/ZnSODs* (*CSDs*) were identified in the *A. trifoliata* genome. Structurally, the phylogeny, intron–exon pattern and motif sequences within these three subfamilies show high conservation. Evolutionarily, segmental/wide genome duplication (WGD) and dispersed duplication form the current *SOD* profile of *A. trifoliata*. Weighted gene coexpression network analysis (WGCNA) revealed the metabolic pathways of nine (69.2%) *SODs* involved in fruit development, among which *AktMSD4* regulates fruit development and *AktCSD4* participates in the stress response. In addition, under the stress of multiple pathogens, six (46.6%) *SODs* were continuously upregulated in the rinds of resistant lines; of these, three *SODs* (*AktMSD1*, *AktMSD2* and *AktMSD3*) were weakly or not expressed in susceptible lines. The results pave the way for theoretical research on *SODs* and afford the opportunity for genetic improvement of *A. trifoliata*.

## 1. Introduction

Reactive oxygen species (ROS), including superoxide radicals (O_2_**^·^**^−^), hydroxyl radicals (**^·^**OH) and hydrogen peroxide (H_2_O_2_), are inevitable byproducts of biological oxidation reactions in plant cells [1], and the production of ROS is further intensified when plants are subjected to biotic and abiotic stresses [2,3,4]. On the one hand, accumulated ROS can act as signaling molecules to regulate the expression of downstream genes to cope with adversity [5]. On the other hand, excessively accumulated ROS can damage plant cell membranes, destroy biological macromolecules, and even cause cell death [1]. In response to the toxic mechanism of ROS, plants have evolved a set of sophisticated antioxidant enzyme systems to maintain ROS homeostasis in cells [6], among which superoxide dismutase (*SOD*) is the first line of defense [7]. Therefore, to explore the molecular mechanism of plant stress tolerance, many scholars have carried out much research on the structure, function and phylogeny of plant *SOD* genes [8,9].

The *SOD* gene family is a class of metal-binding enzymes. Plant *SOD* genes are divided into three groups according to metal cofactors: *iron SOD* (*FeSOD*), *manganese SOD* (*MnSODs*) and *copper/zinc SOD* (*Cu/ZnSOD*) [10]. Another type of *SOD*, *nickel SOD* (*NiSOD*), exists mainly in bacteria, and has not been detected in plants [11]. In terms of the three subfamilies in plants, the subcellular localization of SOD proteins is usually related to the production site of O_2_**^·^**^−^. The compartmentalization of SOD proteins in the cell organelles is extremely important for plant response to oxidation stress induced by abiotic factors and cell signal transformation [12]. Evolutionarily, *FeSODs* and *MnSODs* have ancient origins and are present in bacteria, protozoa, and primitive algae [13]. It is generally believed that *FeSODs* and *MnSODs* have a common ancestor due to their striking similarities [14]. The separate evolution of *FeSODs* and *MnSODs* may be related to changes in oxygen levels on early Earth [13]. For example, the specific Mn/*FeSODs* in a few archaea, such as *Aeropyrum pernix* and *Pyrobaculum calidifontis*, are active with Fe but prefer to bind Mn, especially under aerobic conditions [15,16]. In addition, *Cu/ZnSODs* in plants are considered to have evolved separately from other *SODs* because to date, *Cu/ZnSOD* has not been detected in primitive plants such as *Chlamydomonas* or mosses [13,17].

A large number of reports have confirmed that the inducible expression of *SOD* helps plants respond to abiotic and biotic stresses. For example, the *SOD* activity of salt-tolerant varieties of *Brassica napus* is higher than that of salt-sensitive varieties under salt stress [18]. Under cold stress, the ROS content of cold-tolerant tea tree varieties is higher than that of cold-sensitive varieties, resulting in less damage to the leaves of the former [19]. Furthermore, the plant immune system relies on *SODs* to precisely regulate ROS content, to mediate the immune response to invading pathogenic bacteria. For example, after transferring the tomato *SOD* gene into sugarbeet, the transgenic lines showed strong resistance to oxidative agents as well as the fungus *Cercospora beticola* [20]. In addition to responding to biotic and abiotic stresses, changes in expression and enzyme activity of *SOD* genes during apple fruit ripening suggest that *SOD* plays an important role in fruit development [21,22].

*Akebia trifoliata* (Thunb.) Koidz., is a newly domesticated perennial woody vine with a high commercial development value. The root, stem and unripe fruit of *A. trifoliata* have been used as Chinese medicinal materials for thousands of years [23]. In recent years, *A. trifoliata* has been planted in a large area in Southwest China because of its delicious fruit pulp and high-quality seed oil [24,25]. However, genetic improvement of *A. trifoliata* is still in its infancy due to the short time of development and utilization, especially in terms of resistance to biotic and abiotic stresses. Therefore, improving tolerance of *A. trifoliata* to biotic and abiotic stresses is an urgent task for breeders.

*SOD* and *nucleotide binding sites* (*NBS*) genes are two major means by which plants resist abiotic and biotic stresses [1,26]. To date, the *NBS* gene family in *A. trifoliata* has been systematically identified and analyzed [27], but *SOD* genes have not yet been reported. To speed up theoretical research on adversity stress of *A. trifoliata* to promote progress in commercial exploitation, we systematically identified and analyzed the *SOD* genes of *A. trifoliata* (*AktSOD*), including gene structure, conserved motifs, homology and phylogenetic developmental relationships, based on its published high-quality reference genome. Based on the reported transcriptome data, the expression characteristics of *SOD* genes were also revealed during the fruit development of *A. trifoliata* and critical stage of resisting pathogen invasion, which will help us to initially explore the role of *AktSOD* genes in fruit development and resistance to biotic stress.

## 2. Materials and Methods

### 2.1. Identification and Analysis of AktSOD Genes in A. trifoliata

Genome sequence, protein sequence and annotation files of *A. trifoliata* downloaded from the National Genomics Data Center database under BioProject PRJCA003847 were employed to identify and analyze *SOD* genes. To identify *SOD*-encoding proteins, hidden Markov model (HMM) *Cu*/*ZnSOD* (PF00080) and *Fe-MnSOD* (PF02777 and PF00081) files downloaded from the Pfam database (http://smart.embl.de/smart/batch.pl, accessed on 28 December 2022) were used to filter protein sequences of *A. trifoliata* with the 1 × 10^−5^ e-value parameter [28]. The domains of candidate *AktSOD* proteins were further verified using Conserved Domain Database (CDD) at the National Center for Biotechnology Information (NCBI) (https://www.ncbi.nlm.nih.gov/cdd/, accessed on 3 January 2023). The molecular weight (MW) and theoretical isoelectric point (pI) of proteins were computed via Expasy (https://www.expasy.org/, accessed on 3 January 2023). Subcellular localization was predicted by the online website Plant-mPLoc (http://www.csbio.sjtu.edu.cn/bioinf/plant-multi/, accessed on 3 January 2023). Conserved *AktSOD* protein sequence motifs were analyzed by MEME Suite (https://meme-suite.org/meme/tools/meme, accessed on 5 January 2023). To constberruct the phylogenetic relationship of *AktSOD* protein sequences, after multiple alignments of the *SOD* protein sequences using ClustalW (Kyoto University Bioinformatics Center, Japan) [29], a phylogenetic tree was constructed and drawn by the maximum likelihood method using MEGA 11 software with 1000 bootstrap replicates [29]. Finally, integrated mapping of the *AktSOD* protein structure, conserved motifs and intron–exon pattern was performed using TBtools software (version 1.0876, CJ chen, Guangzhou, China) [30].

### 2.2. Phylogenetic Tree Construction of Plants at Different Evolutionary Nodes

*SOD* protein sequences of *Amborella trichopoda* (basal angiosperm), *Aquilegia coerulea* (basal dicot), *Arabidopsis thaliana* (core dicot), and *Oryza sativa* (monocotyledon) were identified in the same way as those of *A. trifoliata*. The protein sequences of *A. trichopoda* and *A. coerulea* were downloaded from NCBI; those of *A. thaliana* and *O. sativa* were downloaded from Ensembl Plants (http://plants.ensembl.org/index.html, accessed on 6 January 2023). The evolutionary relationship was constructed using IQtree with 1000 bootstrap replicates [31], and the online tool iTOL (https://itol.embl.de/, accessed on 15 January 2023) was used for subsequent visualization [32].

### 2.3. Synteny, Replication and Chromosomal Distribution Analysis of AktSOD Genes

Synteny and replication analysis of *AktSOD* genes was performed using the MCScanX module of TBtools software [30]. Information regarding the physical location of *AktSOD* genes was obtained from the annotation file of the *A. trifoliata* genome. Then, chromosomal distribution and collinearity mapping of *AktSOD* genes were called using the Circos Gene View module of TBtools [30].

### 2.4. Cis-Acting Elements of the AktSOD Gene Family

To predict putative cis-acting elements in *AktSOD* gene promoters, the 2000 bp upstream sequence of *AktSOD* gene coding regions was analyzed using PlantCARE (http://bioinformatics.psb.ugent.be/webtools/plantcare/html/, accessed on 10 January 2023) [33]. A heatmap of the quantity statistics results was prepared using the HeatMap module of TBtools [30].

### 2.5. Expression Analysis of AktSOD Genes in Fruit Development and Rinds at Different Disease Resistance Levels

Transcriptome data for *A. trifoliata* fruit tissues (flesh, rind and seeds) at four developmental stages (young, enlargement, coloring and maturity) were downloaded from the NCBI database (accession IDs: SAMN16551931-33, young stage; SAMN16551934-36, enlargement stage; SAMN16551937-39, coloring stage; SAMN16551940-42, mature stage). Transcriptome data for both the mixed pool of 24 lines exhibiting resistance and another mixed pool of 50 lines exhibiting susceptibility to fungal disease at three different developmental stages were also downloaded from the National Genomics Data Center database under BioProject PRJCA014987. First, transcriptome reads were aligned to the *A. trifoliata* reference genome using HISAT2 software with default parameters [34]. Second, fragments per kilobase of transcript per million fragments mapped (FPKM) values calculated by ESeq2 were used to estimate gene expression levels [35]. Finally, the expression level of *AktSOD* genes was converted to a heatmap by TBtools [30].

### 2.6. WGCNA of the Fruit Development Transcriptome

To investigate the metabolic network in which *AktSOD* genes may participate, 12 transcriptome sets consisting of three fruit tissues at four developmental stages were subjected to weighted gene coexpression network analysis (WGCNA) using the WGCNA shiny plugin of TBtools [30]. A total of 12,016 genes were included in the analysis after filtering out genes with FPKM < 1. Other analysis-related parameters were as follows: R^2^ cutoff = 0.85, module size = 30, and module cutree height = 0.25.

### 2.7. GO Annotation Analysis

Genes in the transcriptome during fruit development were divided into 18 modules by WGCNA. We determined the set of genes expressed in association with the *AktSOD* genes from the module where the genes are located. These gene sets were further subjected to Gene Ontology (GO) annotation analysis via the eggNOG website (http://eggnog-mapper.embl.de/, accessed on 20 January 2023) [36]. The GO Enrichment module of TBtools was used to perform GO enrichment analysis [30].

## 3. Results

### 3.1. Genome-Wide Identification of the SOD Gene Family in A. trifoliata

In this study, a total of 13 *SOD* genes were identified in the *A. trifoliata* genome through HMM analysis and domain validation (Table 1). These 13 *SOD* genes were renamed *AktFSD1*, *AktFSD2*, *AktMSD1–AktMSD4*, and *AktCSD1*–*AktCSD7* according to metal factor type and chromosomal position. The length of the open reading frame of *AktSOD* genes varies greatly, from 2569 bp to 25,161 bp. The results showed that the sequence lengths of the 13 *AktSOD* proteins range from 120 (*AktCSD7*) to 329 (*AktCSD5*) aa, the molecular weights from 12.730 (*AktCSD7*) to 35.181 (*AktCSD5*) kDa, and the isoelectric points from 4.95 (*AktFSD2*) to 9.02 (*AktMSD2*) kDa. According to the prediction of subcellular localization, the products of the two *FeSOD* genes localize to mitochondria (*AktFSD1*) and chloroplasts (*AktFSD2*), those of the 4 *MnSOD* genes to mitochondria, those of the 7 *Cu/ZnSOD* to chloroplasts, and those of *AktCSD2* and *AktCSD7* to the cytoplasm.

### 3.2. Phylogenetic Classification of AktSODs in Plants at Different Evolutionary Nodes

**Figure 1:**
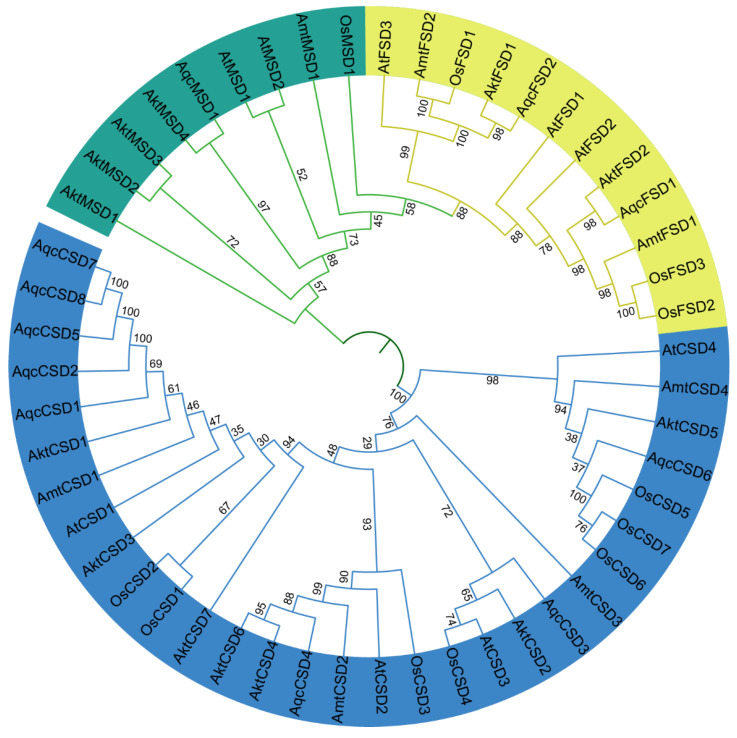
Phylogenetic tree of *SOD* genes from 4 plants: *A. trichopoda*, *A. coerulea*, *A. thaliana* and *O. sativa* (the numbers at nodes indicates bootstrap numbers per 1000 replicates determined by maximum likelihood methods).

To clarify the evolutionary relationship and the classification of *AktSOD* gene subfamilies, 38 reference *SOD* protein sequences from plants at different evolutionary nodes (Table S1), including 7 *SODs* of *A. trichopoda* (basal angiosperm), 11 *SODs* of *A. coerulea* (basal dicot), 9 *SODs* of *A. thaliana* (core dicot), 11 *SODs* of *O. sativa* (monocotyledon) and 13 *SODs* of *A. trifoliata*, were used to construct a phylogenetic tree. In the phylogenetic tree (Figure 1), all 30 *Cu/ZnSOD* genes in the 5 species grouped into one group, all 6 *MnSOD* genes and 12 *FeSOD* genes grouped into another group. Interestingly, the *SODs* of *A. trifoliata* frequently clustered with the *SODs* of *A. coerulea*.

### 3.3. Phylogeny and Conserved Motifs of AktSOD Genes

**Figure 2:**
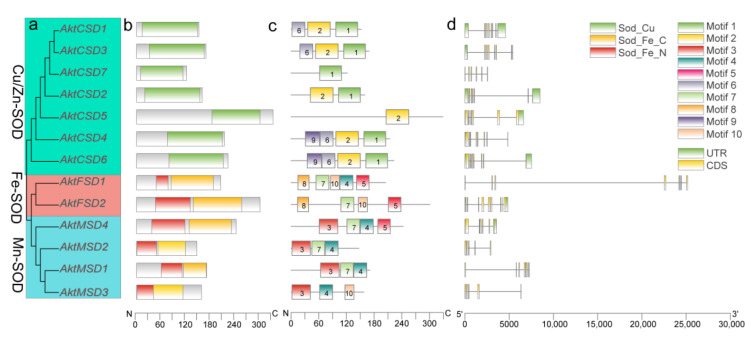
Gene and protein structure analyses of *AktSOD* families. (**a**) Phylogenetic tree of *AktSOD* genes. (**b**) Conserved domains of *AktSOD* proteins. (**c**) Motifs of *AktSOD* proteins. (**d**) Exon–intron structures of *AktSOD* genes.

The 13 *AktSODs* were divided into two groups in the phylogenetic tree (Figure 2a): 7 *Cu/ZnSODs* were classified into one group; the other 6, consisting of 2 *FeSODs* and 4 *MnSODs*, were classified into another group. Identification of conserved domains indicated that the 2 *Akt-FSODs* and 4 *Akt-MnSODs* have similar domain compositions, with both containing an Sod_Fe_C and Sod_Fe_N domain. In contrast, *Akt-Cu/ZnSODs* have only 1 Sod_Cu domain (Figure 2b). Motif analysis further revealed similarities and differences in the protein sequences of the three subfamilies of *AktSODs*. A total of 10 conserved motifs were identified in the 13 *AktSODs* (Figure 2c, Table S2): motifs 1, 2, 6, and 9 are only present in *Akt-Cu/ZnSODs*; motifs 4, 5, 7, and 10 are common to *Akt-FeSODs* and *Akt-MnSODs*; motif 8 exists only in *Akt-FeSODs*; and motif 3 is unique to *Akt-MnSODs*. Structurally, the coding sequences of the 13 *AktSODs* are separated by multiple introns, with the number of introns ranging from 4 to 8 (Figure 2d; Table 1).

### 3.4. Chromosomal Distributions and Duplication Types of AktSODs

**Figure 3:**
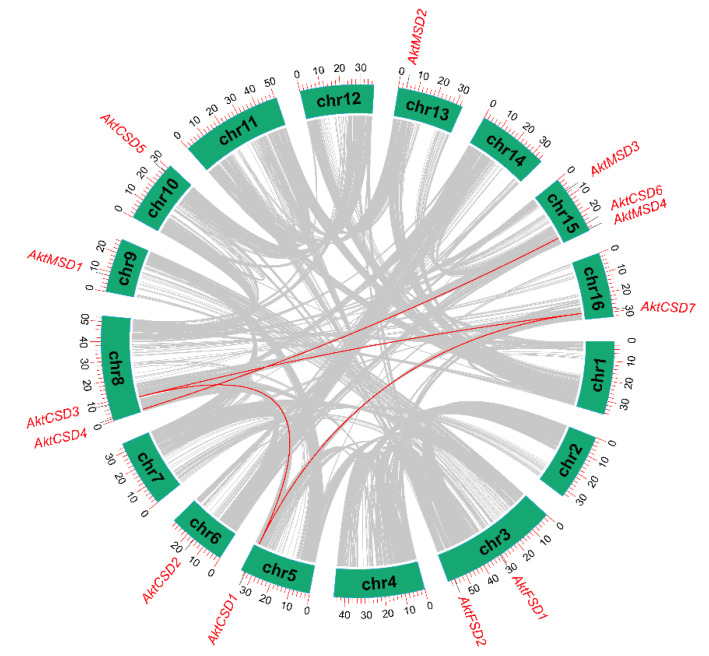
Chromosomal localization and interchromosomal synteny of *AktSODs*. Red lines in the inner circle represent syntenic gene pairs from WGD/fragment duplications.

The chromosomal physical locations of the 13 *AktSODs* are depicted in Figure 3. Among the 16 chromosomes of *A. trifoliata*, 9 chromosomes harbor 13 *AktSOD* genes. Chromosomes 5, 6, 9, 10, 13 and 16 each carry an *SOD* gene, chromosomes 3 and 8 both carry 2 *SOD* genes, and chromosome 15 carries the remaining 3 *SOD* genes. Gene duplication analysis of the *AktSOD* family revealed dispersed duplication in 8 *SOD* genes; the other 5 *SOD* genes experienced segmental/wide genome duplication (WGD) (Table 1; Figure 3).

### 3.5. Detection of Cis-Acting Elements in the Promoter Sequence of AktSODs

**Figure 4:**
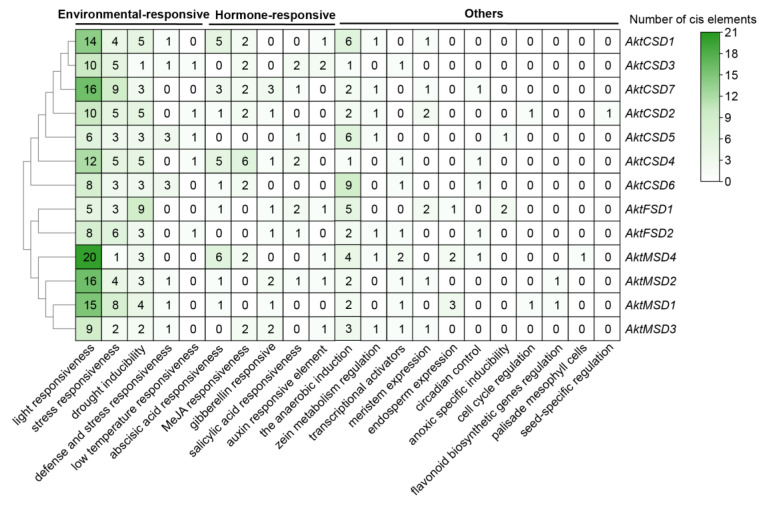
Analysis of cis-regulatory elements in the 2 kb region upstream of the transcription start site of *AktSODs*.

Statistical results for putative cis-acting elements showed a large number of environmental and hormone-responsive elements to be distributed throughout the promoter region of *AktSODs* (Figure 4; Table S3). All *AktSODs* contain at least one light-responsive, stress-responsive and drought-inducible element. The number of light-responsive elements is greatest; *AktFSD1* has the lowest number, at 5; *AktMSD4* has the highest number, at 20. In addition, 5 plant hormone-responsive elements, including for abscisic acid (ABA), methyl jasmonate (MeJA), gibberellin (GA), salicylic acid (SA) and auxin, were detected in the promoter region of *AktSODs,* with the distribution varying. For example, *AkCSD5* has only one hormone-responsive element, namely, the TCA element, which is annotated as responsive to salicylic acid (Table S3). *AkCSD4*, *AkCSD7*, *AkFSD1*, and *AkMSD2* contain four types of hormone-responsive elements.

### 3.6. Expression Analysis of AktSODs in Different Tissues of A. trifoliata Fruit

**Figure 5:**
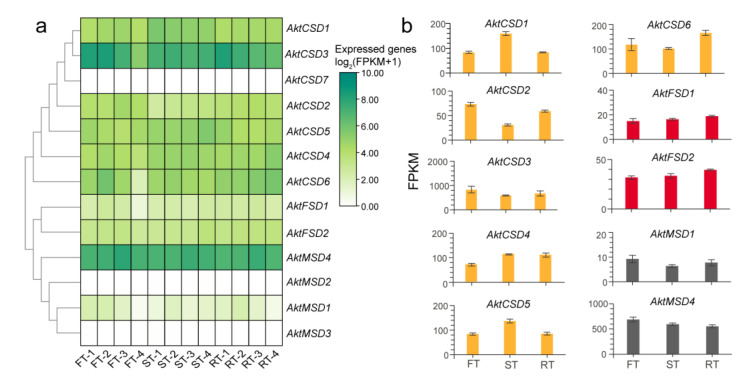
Expression levels of 13 *AktSODs* in different tissues and developmental stages (FT: flesh, ST: seed, RT: rind, 1: young stage, 2: enlargement stage, 3: coloring stage, 4: mature stage). (**a**) Heatmap of expression of 13 *AktSODs* in three fruit tissues at four stages. (**b**) Total expression of *AktSODs* in three fruit tissues contains the total FPKM of each fruit tissue in its four development stages. The error bars of the column represent the standard deviation of FPKM in the four development stages. The different colors of the column represent three subfamilies of *AktSODs*.

Based on the expression analysis of *AktSODs* using flesh, seed and rind tissues, the expression of 12 *AktSODs* was detected, with the levels of most being similar in the flesh, seed and rind tissues at four developmental stages (young, enlargement, coloring, and mature stage) (Figure 5a; Table S4). Only the expression of *AktCSD7* could not be detected in any of the samples. In contrast, the expression level of *Cu/ZnSODs* was higher than that of the other two subfamilies of *SODs*; *AktCSD3* showed the highest expression level. Two *FeSODs* (*AktFSD1* and *AktFSD2*) showed persistent low expression levels during the development of flesh, seed and rind tissues. Among the 4 *MnSODs*, *AktMSD4* was continuously expressed at a high level, but *AktMSD2* and *AktMSD3* were only detected in trace amounts in some fruit tissues (Table S4). By comparing the total expression of *AktSODs* in flesh, seed and rind tissues, higher expression levels of *AktCSD2*, *AktCSD3*, *AktMSD1* and *AktMSD4* in flesh were observed; *AktCSD1, AktCSD4* and *AktCSD5* showed slightly higher expression levels in seeds, and only *AktCSD6* displayed higher expression in rinds (Figure 5b). Overall, expression levels of *AktFSD1* and *AktFSD2* were extremely similar in the three fruit tissues (Figure 5b).

### 3.7. Metabolic Regulatory Network Involved in AktSODs in Fruit Development

We performed WGCNA on 12 transcriptome datasets during fruit development of *A. trifoliata*, and found that *AktSOD*s participate in the metabolic regulatory network. The genes expressed in the transcriptomes after filtering low-expression genes were divided into 18 modules (Figure S1); 9 *AktSODs* were assigned to 6 modules, and the remaining 4 *AktSODs* were excluded due to low expression or no expression in fruit tissues. *AktFSD1*, *AktFSD2* and *AktCSD6* were assigned to the same module. *AktCSD1* and *AktCSD2* were assigned to another module, and the remaining 4 *AktSODs* were assigned to four different modules.

**Figure 6:**
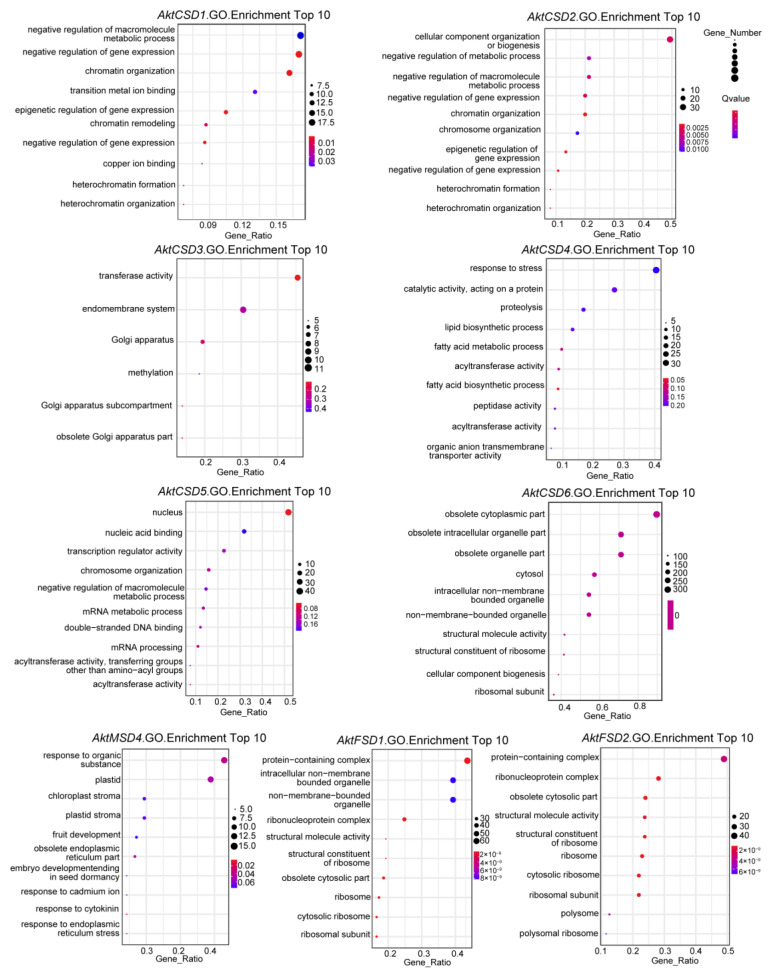
GO enrichment analysis of gene sets associated with *AktSOD* expression.

We further focused on the set of genes expressed in association with the 9 *AktSODs* from the module in which the *AktSODs* are located (Table S5). GO enrichment analysis was performed on the 9 *AktSOD*-associated gene sets, and the results are illustrated in Figure 6. The *AktSOD* genes assigned to the same module collectively participate in a large number of identical metabolic networks, but there are also differences. For example, the gene sets associated with the expression levels of *AktCSD1* and *AktCSD2* were enriched in the negative regulation of gene expression and chromatin organization. Conversely, *AktCSD1*-related genes were more assigned to the negative regulation of macromolecule metabolic processes, unlike *AktCSD2*. The gene sets associated with the expression levels of *AktFSD1*, *AktFSD2* and *AktCSD6* were all enriched in the obsolete cytoplasmic part and ribosomal subunit, and the metabolic network involving *AktFSD1* and *AktFSD2* was more similar than that of *AktCSD6*. In addition, it is worth noting that the gene set associated with the expression level of *AktCSD4* is associated with the response to stress, and that the gene set associated with the expression level of *AktMSD4* is related to fruit development.

### 3.8. Differential Expression of AktSODs in Resistant and Susceptible Rinds

**Figure 7:**
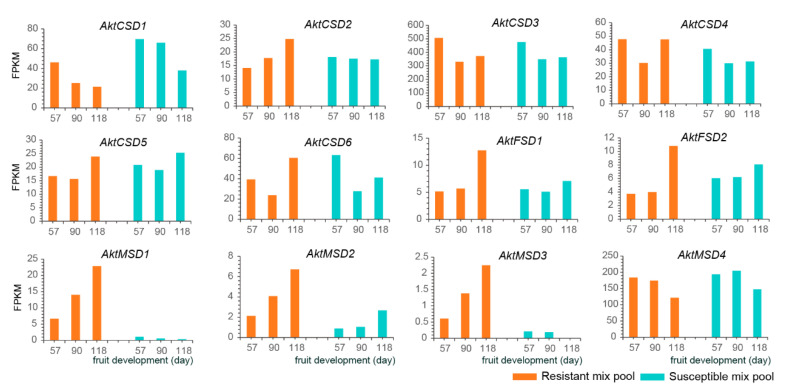
Expression-level analysis of 13 *AktSODs* in resistant and susceptible rinds.

Transcriptome data for rind tissues with different disease resistance levels were used to explore the expression of *AktSODs* in response to various pathogenic bacteria. By comparing the expression levels of *AktSODs* in resistant rinds (a mixed pool of 24 lines) and susceptible rinds (a mixed pool of 50 lines), it was found that almost all *AktSODs* were significantly expressed in resistant or susceptible rinds, except for *AktCSD7* (Figure 7; Table S6). Among the 12 *AktSODs*, *AktCSD2*, *AktFSD1*, and *AktFSD2* showed more drastic upregulation in resistant rinds during the period of rapid fruit expansion than in susceptible lines. In addition, the three *MnSOD*s (*AktMSD1*, *AktMSD2* and *AktMSD3*) were rapidly upregulated in resistant rinds, with extremely weak or even no expression in susceptible rinds.

## 4. Discussion

*A. trifoliata* is a newly domesticated fruit tree, horticultural crop and potential oil crop [37]. Abiotic and biotic stresses are the main factors that cause serious declines in the fruit yield and quality of artificially cultivated *A. trifoliata*. *SOD* genes have been reported to play a crucial role in biotic stress, abiotic stress and fruit growth and development. In our current research, the reference genome and transcriptome data available for *A. trifoliata* provided an opportunity to explore the function of *AktSODs* and their expression in response to fruit development and biotic stress.

### 4.1. The AktSOD Family Is Highly Conserved during Evolutionary Adaptation

In the current study, we identified 13 *SOD* genes in the *A. trifoliata* genome, including 2 *FeSODs*, 4 *MnSODs* and 7 *Cu/ZnSODs*. Similar to most angiosperms, the number of *AktSOD* family members is highly conserved in evolution, with almost no large-scale expansion, such as the 7 *SOD* genes in barley [38] and *A. thaliana* [39], the 9 *SOD* genes in tomato [40], the 10 *SOD* genes in grapevine [41], the *13 SOD* genes in maize [42], and the 26 *SOD* genes in wheat [43]. The three *AktSOD* subfamilies are also highly conserved with regard to gene structure. *Akt-FeSODs*, *Akt-MnSODs* and *Akt-Cu/ZnSODs* include different motifs in their protein sequences, with a bias observed (Figure 2c). Gain and loss of introns/exons is a driving force of gene evolution [44]. Unlike the *SOD* genes of the tea plant [45] and rapeseed [46], the number of introns/exons of *AktSOD* genes has changed only slightly, especially in the same subfamily (Table 1). The intron–exon pattern of *AktSODs* also indicates their conservation in the evolutionary process.

Evolutionarily, different types of gene duplication may occur along different evolutionary trajectories, and may be preserved in a biased manner in different types of gene families [47]. In *A. trifoliata*, 5 (38.5%) *AktSODs* derived from genome-wide duplication events, among which 4 pairs of homologous *Cu/ZnSODs* show collinearity (Figure 3); the remaining 8 (61.5%) *AktSODs* derived from dispersed duplications (Table 1). It is worth noting that two WGD events occurred in the *A. trifoliata* genome for adaptation to drastic changes in the environment [48]. In *A. trifoliata*, the strong WGD only expanded *Cu/ZnSOD* genes by a few copies, whereas *FeSODs* and *MnSODs* appear to have been unaffected by WGD. This evolutionary evidence further demonstrates that *AktSODs* are highly conserved.

### 4.2. AktSODs Are Largely Functionally Diverse and Widely Involved in A. trifoliata Fruit Development

Overall, the physical properties of proteins play an important role in determining the biochemical function of the molecule [49]. Physical characterization of *SOD* proteins showed extensive differences in protein sequence length, isoelectric point and molecular weight in the 13 *AktSOD* proteins (Table 1). Further focusing on cis-elements in their promoter regions revealed a large number of light-responsive and hormone-responsive elements distributed in the 13 *AktSODs* (Figure 4). The abundant environmental and plant hormone-responsive elements in *AktSOD* promoters may be related to different roles or regulatory mechanisms in response to biotic and abiotic stresses. In addition, great variation in number and type exists, and some elements responding to metabolism and gene expression are specific to some *SOD* genes. For example, 6 (46.15%) *AktSODs* have meristem expression responsive cis-elements, but 5 (38.16%) *AktSODs* lack transcriptional activator responsive cis-elements (Figure 4). In general, differences in the structure of *AktSODs* may cause changes in protein function.

Differences in expression levels and tissue specificity of a gene family are often associated with functional differentiation [50]. Gene expression analysis showed 12 (92.31%) of the *AktSODs* to be involved in fruit growth and development. *AktCSD3* and *AktMSD4* were persistently expressed at high levels in flesh, seed and rind tissues, whereas *AktMSD2* and *AktMSD3* showed spatiotemporally specific expression during fruit development. Such specific expression of *AktSODs* indicates that they are involved in different physiological activities.

In previous studies, it has been reported that the main function of *SOD* genes is to respond extensively to various abiotic stresses [45], and increasing *SOD* activity is an effective way to improve stress resistance in plants [51,52]. However, in the process of fruit development, ROS is continuously released during vigorous metabolic activities [53], and the surface of the fruit is also attacked by various pathogenic bacteria, so the *SOD* genes also play an important role in the process of fruit development. In the current study, WGCNA of transcriptome data further revealed that these *AktSODs* participate in distinct metabolic networks during fruit development. First, the 9 (69.23%) *AktSODs* included in the analysis were assigned to 6 modules, and expression levels of 5 (39.46%) *AktSODs* were associated with other *SOD* genes, suggesting a cooperative division of labor between *AktSOD* genes. Second, the gene sets associated with *AktSODs* were enriched in different molecular function, biological process and cellular component networks, also confirming that the metabolic network pathways regulated by *AktSODs* are different. Through enrichment analysis, we also inferred that *AktCSD4* is extremely likely to be involved in the stress response, and that *AktMSD4* may be involved in the regulation of fruit development because the associated expression gene set was enriched in the corresponding pathway.

### 4.3. Three MnSODs May Play an Important Role in Resisting Invasion of Pathogenic Bacteria

The relationship between *SODs* and plant disease resistance has been extensively studied due to its focus on the regulation of endogenous ROS [54]. A large number of reports indicate that some *SOD* genes can enhance the resistance of plants to specific pathogens. For example, tobacco has been transformed with a *Cu/ZnSOD* gene from *Spinacia oleracea*, not only improving its tolerance to water stress but also its resistance to *Pseudomonas syringae* pv. *tabaci* [55]. Another study reported that after a *MnSOD* gene derived from oil radish was transferred to broccoli, the transgenic lines showed a higher resistance to downy mildew [56]. Comparing expression levels of *SOD* genes in the rinds of various resistant and susceptible lines, we found that some *SOD* genes may be induced by pathogenic bacteria under natural conditions. For instance, 3 *Akt-MnSOD* genes, *AktMSD1*, *AktMSD2* and *AktMSD3*, were expressed at very low levels or even not expressed during the development of fruit tissues and in susceptible rinds, but they were stably upregulated in resistant rinds (Figure 7). Such inducible expression of these 3 *MnSOD* genes in resistant rinds suggests that they might play an important role in the defense against pathogenic bacteria.

## 5. Conclusions

In this bioinformatics study, a total of 13 *SODs* classified into three categories were identified in the reference genome of *A. trifoliata*. The phylogeny, motif composition, intron–exon pattern and replication type of the *AktSODs* all indicate that these *SODs* of *A. trifoliata* are extremely conserved in the evolutionary process, and that only a few copies have undergone expansion by segmental duplication/WGD and dispersed duplication. WGCNA of the transcriptome of *A. trifoliata* fruit revealed the metabolic network in which *AktSOD* genes may be involved during fruit development, with *AktMSD4* being involved in responding to stress and *AktCSD4* possibly regulating fruit development. Comparing the expression of *AktSOD* genes in resistant and susceptible rinds, it was found that *AktMSD1*, *AktMSD2* and *AktMSD3* are induced by pathogens and may be involved in defense against these organisms. Overall, this study lays the foundation for improving biotic and abiotic stress responses of *A. trifoliata* and provides information on the regulation of *AktSOD* gene expression, especially during fruit development.

## Figures and Tables

**Table 1:** Characteristics of the *SOD* gene families identified in the *A. trifoliata* genome.

Name	Gene ID	SOD Type	AA	pI	MW (kDa)	Number of Exon	Number of Intron	Duplication Type	Subcellular Localization
*AktCSD1*	*EVM0009170*	Cu/ZnSOD	152	5.66	15.362	8	7	WGD/Segmental	Chloroplas.
*AktCSD2*	*EVM0011294*	Cu/ZnSOD	160	6.79	23.238	7	6	Dispersed	Chloroplast, Cytoplasm
*AktCSD3*	*EVM0015107*	Cu/ZnSOD	169	5.27	17.118	8	7	WGD/Segmental	Chloroplast
*AktCSD4*	*EVM0004711*	Cu/ZnSOD	214	6.39	21.895	8	7	WGD/Segmental	Chloroplast
*AktCSD5*	*EVM0014685*	Cu/ZnSOD	329	5.36	35.181	7	6	Dispersed	Chloroplast
*AktCSD6*	*EVM0003060*	Cu/ZnSOD	223	6.79	23.238	8	7	WGD/Segmental	Chloroplast
*AktCSD7*	*EVM0005862*	Cu/ZnSOD	120	6.2	12.730	6	5	WGD/Segmental	Chloroplast, Cytoplasm
*AktFSD1*	*EVM0017725*	FeSOD	205	7.11	24.065	8	7	Dispersed	Mitochondrion
*AktFSD2*	*EVM0016720*	FeSOD	301	4.95	34.734	9	8	Dispersed	Chloroplast
*AktMSD1*	*EVM0002565*	MnSOD	171	7.89	18.538	7	6	Dispersed	Mitochondrio
*AktMSD2*	*EVM0008804*	MnSOD	147	9.02	16.154	5	4	Dispersed	Mitochondrion
*AktMSD3*	*EVM0020914*	MnSOD	158	7.78	17.433	5	4	Dispersed	Mitochondrion
*AktMSD4*	*EVM0011812*	MnSOD	243	8.38	27.293	6	5	Dispersed	Mitochondrion

Abbreviations: AA, the number of amino acids; pI, isoelectric point; MW, theoretical subunit size of the proteins.

## Data Availability

All data analyzed during this study are included in the manuscript and Supplementary Information files. Genome sequence files of *A. trifoliata* were downloaded from the National Genomics Data Center database under BioProject PRJCA003847. Transcriptome data for *A. trifoliata* were downloaded from the NCBI database under accession numbers PRJNA671772, SAMN16551931–33, SAMN16551934–36, SAMN16551937–39, SAMN16551940–42 and the National Genomics Data Center database under BioProject PRJCA014987.

## Supplementary Materials

The following supporting information can be downloaded at https://www.mdpi.com/article/10.3390/antiox12030726/s1, Figure S1: WGCNA of the fruit developmental transcriptome; Table S1: *SOD* sequences in four different evolutionary node species; Table S2: The information of identified 10 motifs in AktSOD proteins; Table S3: Detailed information of cis-acting elements in the 2000 bp region of *SOD* gene coding regions; Table S4: The expression level of 13 *SOD* genes in the fruit transcriptome of *A. trifoliata*; Table S5: List of genes expressed in association with *AktSOD* genes in the same module; Table S6: The expression level of *SOD* genes in resistant and susceptible rinds of *A. trifoliata.*
