# Supplementary figures and images for: Genome-Wide Identification of Superoxide Dismutase and Expression in Response to Fruit Development and Biological Stress in Akebia trifoliata: A Bioinformatics Study
Source: Antioxidants (Basel). 2023 Mar 15;12(3):726. doi: 10.3390/antiox12030726 (PMC10045841; doi:10.3390/antiox12030726)

Supplementary figure

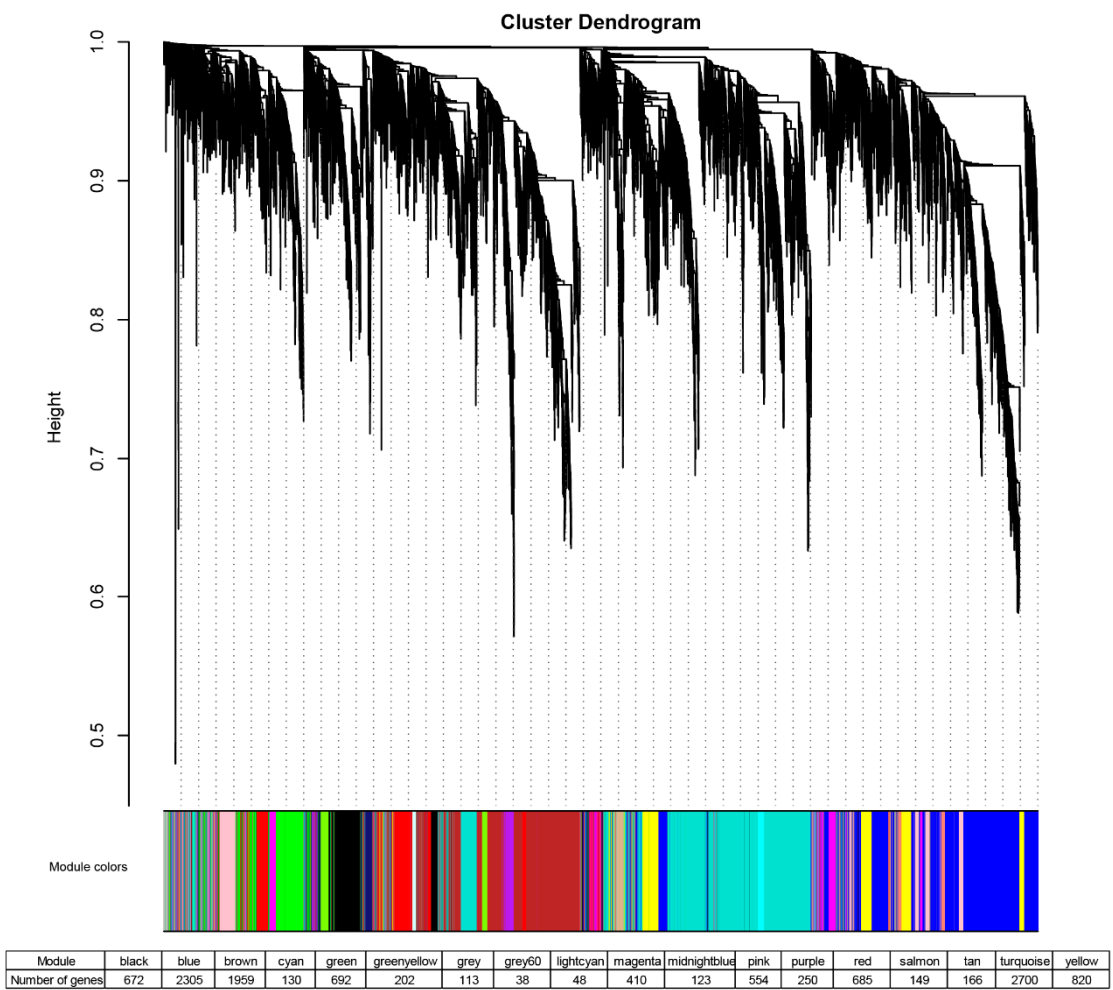

Supplementary Figure S1. WGCNA of fruit developmental transcriptome.

Supplement: Supplementary file 1 [file antioxidants-12-00726-s001.zip › supplementary Figure S1.pdf]
